# Graphene oxide:Fe_2_O_3_ nanocomposites for photodetector applications: experimental and *ab initio* density functional theory study

**DOI:** 10.1039/d3ra00174a

**Published:** 2023-02-21

**Authors:** David O. Idisi, Chinedu C. Ahia, Edson L. Meyer, Joseph O. Bodunrin, Evans M. Benecha

**Affiliations:** a Fort Hare Institute of Technology, University of Fort Hare Private Bag X1314 Alice 5700 South Africa Didisi@ufh.ac.za; b Department of Physics, CSET, University of South Africa Private Bag X6, Florida Science Campus, Christiaan de Wet and Pioneer Avenue, Florida Park, Florida 1710 Johannesburg South Africa

## Abstract

In this report, a GO:Fe_2_O_3_ nanocomposite was synthesized using a one-step covalent attachment approach using a sol–gel technique. The optical absorbance, photoconductive, photo-capacitive, and electrical properties were obtained using spectroscopy, and current–voltage (*I*–*V*) measurements. An enhanced optical absorbance with corresponding band gap reduction is observed when Fe_2_O_3_ nanoparticles are incorporated in GO. A corresponding enhanced photoconductance in the order of ×10^1^ was observed due to the impact of band gap narrowing. The enhanced photoconductivity and photo-capacitance can be attributed to energy and charge transfer between GO and Fe atoms, leading to the generation of photo-induced excitons. Density function theory calculations indicate increased charge transfer when GO is doped with Fe–O atoms, which is consistent with experimental data. The observed results could potentially enable the use of GO:Fe_2_O_3_ nanocomposites for photodetectors and other optoelectronic applications.

## Abstract 2

GO/ GO: Fe_2_O_3_ nanocomposite fabricated on SiO_2_/ Si wafer substrate with Ag electrical contacts using drop cast technique.

## Graphical abstract

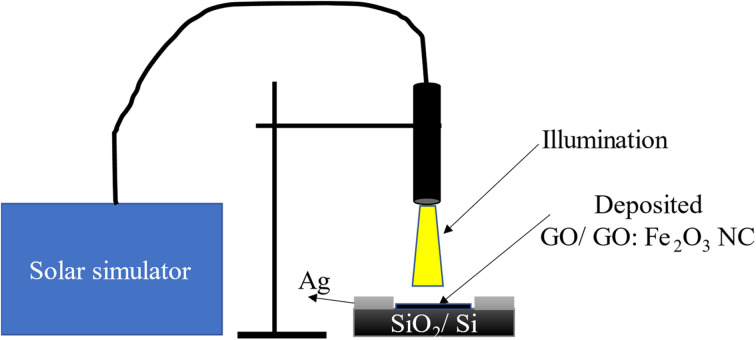

## 1 Introduction

Photoconductivity and photo-capacitance are properties that enable the fabrication of devices for detecting light and charge storage. The principle behind such devices involves the conversion of electric light signals into photocurrent and photo-charges (in the case of charge storage). These photocurrents and photo-charges could be useful for photodiodes, phototransistors, solar cells and optical communications applications.^1–5^

Graphene oxide (GO), which is a chemical derivative of graphene, offers enormous promise for various scientific applications due to its outstanding electronic properties.^6,7^ Due to the hydrophilic nature of GO, its properties can be easily tuned by attaching metals/oxides to the edge, basal planes and its surface.^8^ The electrical conductance property of GO can be useful for organic solar cells and light-emitting diode (LED) applications.^9,10^ Moreover, photo-sensing, which forms part of optoelectronics, has been a subject of investigation in the last decade. Of particular interest is the photo-conductance properties of GO which could be exploited for photodetectors and photovoltaic applications.^11^ The tunability of band gap and high absorbance offered by GO is essential for optoelectronic applications.

Although the experimental realization of photoconductance of GO has been realized,^12^ the feasibility of graphene for photodetector devices still suffers a setback from its zero-band gap nature. The on–off current efficiency of field effect transistors still poses challenges.^13^ Hence, using graphene for photo-sensing applications still requires attention.

Attempts by different researchers to improve the photo-conductive properties of GO have been made. For instance, Liang *et al.*^14^ explored the photo-conductive behavior of GO/rGO composite films. Their result showed a high proto-conductive response and photovoltaic response in the range 0.017–0.014 V. Najla^15^ explored the electrical and photo-conducting properties of Al/graphene oxide nanocomposite using NiO as the dopant. The result showed an increased reversed current with increased photon illumination. Recently, Naveed *et al.*^16^ synthesized α-Fe_2_O_3_/graphene nanocomposite where the photoconductive properties were explored as anode material for photoelectrochemical water splitting applications. Their results showed great promise for enhancing the efficiency of hydrogen fuel production.

Meanwhile, density functional theory calculations have offered a means of understanding the conduction mechanism and doping effect on the properties of heterostructures. The process of synthesis of nanomaterials does not account for the control location of dopants/functionalizers. However, DFT offers the possibility of studying the optimal location of dopants for enhanced properties of desired heterostructures. Hence, the incorporation of DFT calculations in the current study provides insight into the charge transfer mechanism as well as optimal dopant sites for a detailed understanding of the properties of Fe–O in the GO matrix, which is lacking in most of the previously reported studies.

GO:Fe_2_O_3_ nanocomposite has not been widely explored for optoelectronics applications, especially with photo-capacitive properties. In the current study, GO:Fe_2_O_3_ nanocomposite was synthesized for photoconductive response application. Hence, the relationship between the illumination of GO/Fe_2_O_3_ nanocomposite with white light of different wavelengths, and the current–voltage characteristics have been considered in this study. Our results showed great promise toward applications in photodetectors and solar radiation detectors.

## 2 Experimental details

High purity iron(iii) chloride hexahydrate (FeCl_3_·6H_2_O) and ammonia hydroxide (NH_4_OH) were commercially acquired from Sigma-Aldrich. GO was synthesized using Hummers' method as previously reported.^17^ Details of the experimental procedure for GO are available elsewhere in our previous report.^18^ Fe_2_O_3_ nanoparticles were synthesized by co-precipitation technique as reported by Sankadiya *et al.*^19^ For the typical synthesis of Fe_2_O_3_ nanoparticles, 6 g of (FeCl_3_·6H_2_O) was dissolved in a 150 mL beaker forming a solution with deoxidized water. A solution of 50 g of NH_4_OH (30 mL) and 20 mL of deoxidized water was prepared in another beaker. The NH_4_OH solution was added to the (FeCl_3_·6H_2_O) solution in a dropwise manner while being steadily stirred till the pH = 8 and a precipitate was attained. The obtained precipitate was filtered and washed using centrifuge system several times. The precipitate was air dried for approximately 13 hours and calcinated at 400 °C for 4 hours to reduce the impact of impurities resulting reddish-brown powder. A portion of the Fe_2_O_3_ powder was dissolved in deoxidized water and mixed with GO in a ratio of 1 : 1 and sonicated for 30 minutes. The obtained nanocomposite was air-dried, and the powder was collected for further characterization.

**Fig. 1:**
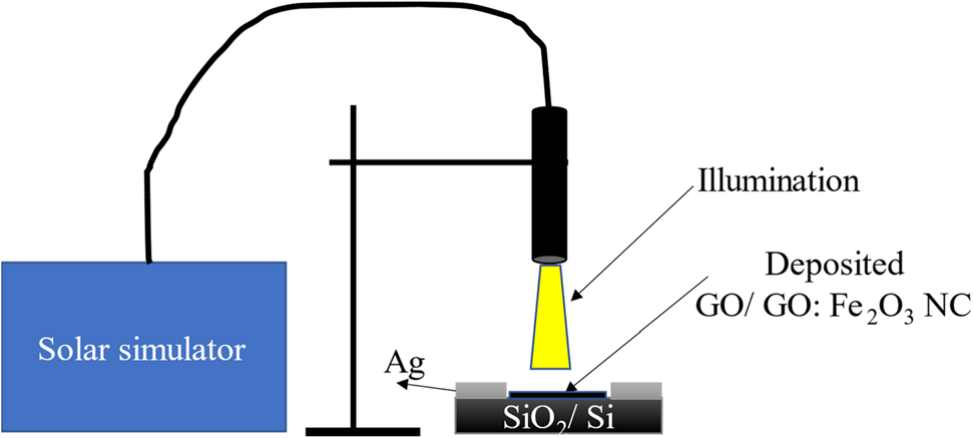
Schematics of the process of photo-conductivity characterization.

The process of fabrication and characterization of the device involved the deposition of GO and GO:Fe_2_O_3_ NC on SiO_2_/Si substrate as depicted in the schematics shown in Fig. 1. Firstly, the uniquely cut SiO_2_/Si wafer (1 × 1 cm^2^) was dissolved in both deoxidized and ethanol to remove impurities. The obtained unfunctionalized and Fe_2_O_3_ functionalized GO were dissolved in methanol and subsequently sonicated for 15 min for homogeneity. The dissolved GO and GO:Fe_2_O_3_ NC was coated on the SiO_2_/Si wafer and allowed to air-dry for 12 h. Silver gel was used as an electrical contact and allowed to air-dry for 12 h.

### 2.1 Characterization techniques

XRD measurement was performed using a Rigaku X-ray diffractometer (*λ* = 1.54 nm). Transmission electron microscopy (TEM) and scanning electron microscopy (SEM) were measured using JEOL JEM 2100 transmission electron microscopy (accelerating voltage 200 kV) and field emission SEM (FESEM) JSM-7800F (accelerating voltage 5.0 kV) from Jeol Ltd, respectively. Photo-conductance and photo-capacitance measurements were performed using Keithley 6497 as the current–voltage source, whereas ASAHI HAL-320 solar simulator (output wavelength 350–1800 nm) was the light source. The diameter of the light source was varied to cover the entire surface of each of the films. The voltage sweep was set in the range −1 to +1 V for photocurrent measurement whereas, for photo-capacitance, 0–2 V with frequency 1 kHz was used. All the measurements were performed at room temperature.

### 2.2 Computational details

The electronic properties of Fe–O doped GO and undoped GO were calculated using the Cambridge Serial Total Energy Package (CASTEP).^20^ The electron–electron exchange effects were performed using Perdew–Burke–Ernzerhof generalized gradient approximation (PBE-GGA) functional,^21^ while the valence core interactions were represented using the Vanderbilt ultrasoft pseudopotentials.^22^ Geometry optimization was performed using 4 × 4 graphene supercell containing 1-OH and 1-O functional groups, with lattice parameter *a* = 9.84 Å, *b* = 9.84 Å and a vacuum space of 15 Å in the *c*-direction to minimize interlayer interactions, while the Fe–O atoms were placed as adatoms on the graphene surface. The wave function was represented using expanded plane wave basis sets, with a well converged plane wave cut off energy of 800 eV, while sampling of Brillouin zone was done using optimized 8 × 8 × 2 *k*-points. A denser *k*-point grid of 10 × 10 × 2 was used to calculate the electron density difference and density of state calculations to correctly account for electron transport and electronic properties of rGO and Fe–O doped rGO. The adatomic siting of the Fe–O atoms on the GO matrix was based on our previous report,^23^ where enhanced magnetization was observed for the Fe atom sitting at an adatom site, as opposed to the interstitial or substitutional site.^23^ Different doping configurations, each with a varying number of Fe–O pairs, were considered (Fig. 7) to account for the effect of dopant concentration on the electronic properties of GO.

## 3 Results and discussions

**Fig. 2:**
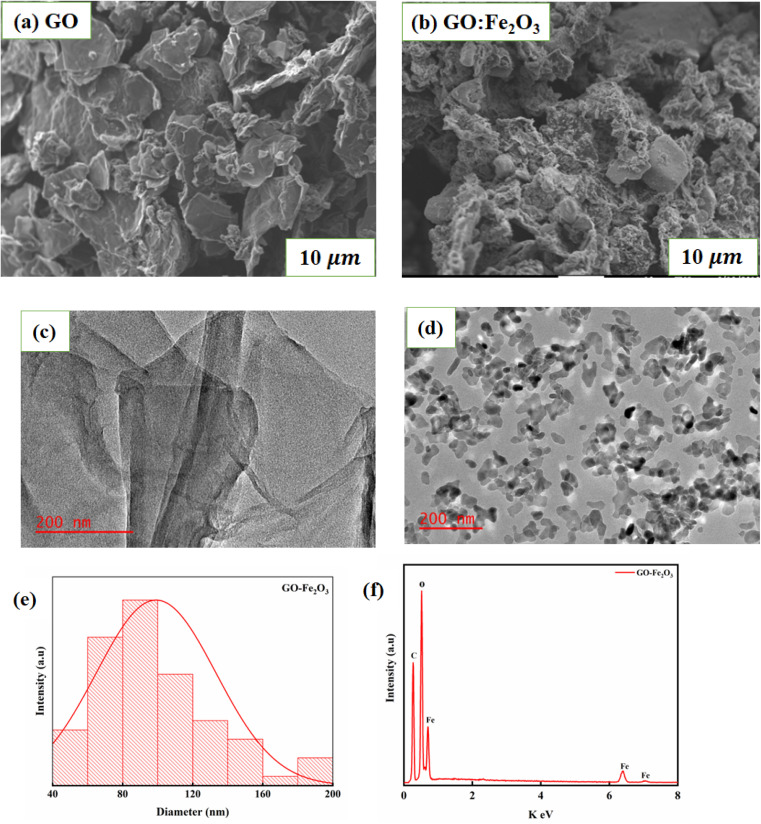
Morphology and particle analysis of GO and GO:Fe_2_O_3_ nanocomposite (a) and (b) SEM (c) and (d) TEM images and (e) and (f) particle size distribution and EDX spectra from TEM measurements.

Fig. 2(a) and (b) shows the SEM images of GO and GO:Fe_2_O_3_ composite where the flake-like sheets of GO are present. The sheets seem to overlap each other signifying weak agglomeration and/or coalescence sheets whereas the case of GO:Fe_2_O_3_ composite shows a slight modification of the sheets. The spheres that are associated with Fe_2_O_3_ are less prominent. The appearance may be attributed to the overlap or the Fe spheres sitting on top of the GO sheets and is consistent with the XRD spectra. Fig. 2(c) and (d) show the TEM images of GO and GO:Fe_2_O_3_ nanocomposite where the sheets of GO are indicated as expected. The particles representing Fe_2_O_3_ are distributed evenly on the surface of GO with a high level of agglomeration of the Fe_2_O_3_ particles. Fig. 2(e) and (f) shows the particle size distribution and EDX spectra analysis of GO:Fe_2_O_3_ nanocomposite from TEM measurements. The distribution indicates most of the particle sizes were in the range 80–100 nm, which suggests a monomodal particle size distribution^24^ and are in proximity of the crystallite size (see Table 1), which was obtained from XRD. The EDX spectra on the other hand show the presence of C, O and Fe in the sample as expected.

**Table 1:** XRD analysis showing the crystallite size and *D*-spacing using 001 and 002 peak positions for GO and GO:Fe_2_O_3_ nanocomposite

Samples	Peak position (2*θ* (°))	FWHM (a.u.)	Crystallite size (nm)	*D*-Spacing (nm)	Lattice constant (Å)
GO	13.2 (001)	2.6181	31.92	6.71	
	23.0 (002)	4.5912	18.45	7.73	
GO:Fe_2_O_3_	13.4 (001)	1.1751	71.12	6.61	*a* = *b* = 5.01
					*c* = 13.67
	24.3 (002)	1.1840	71.71	7.33	

**Fig. 3:**
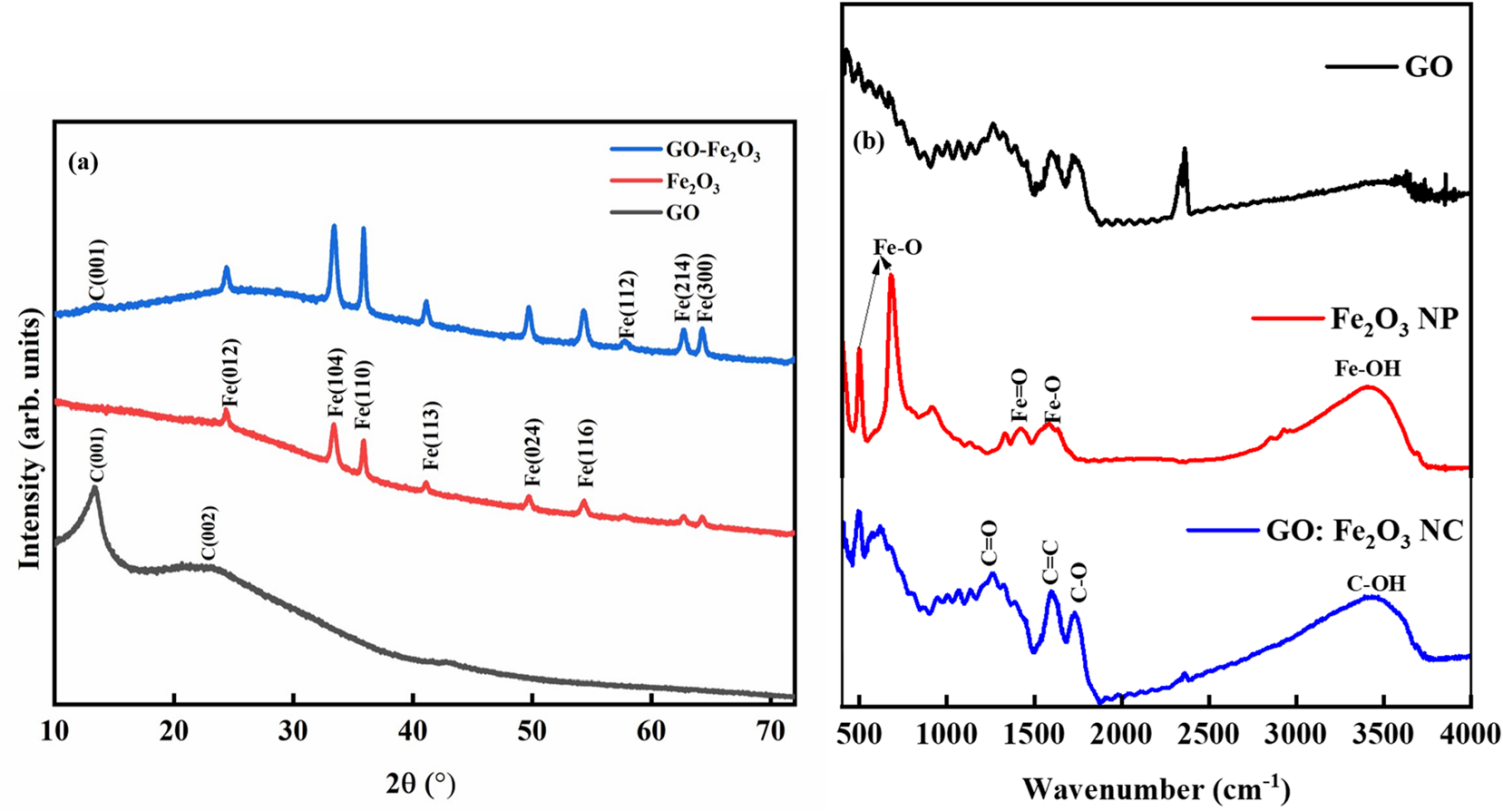
Structural and bonding properties of GO and GO:Fe_2_O_3_ nanocomposite (a) XRD pattern (b) FTIR spectra.

Fig. 3(a) shows the XRD spectra for GO, Fe_2_O_3_ and GO:Fe_2_O_3_ composite where the prominent peaks are located at 2*θ* ∼24.3°, 33.3°, 35.8°, 41.1°, 49.7°, 54.3° and 57.8°. The peaks are assigned to Fe (012), Fe (014), Fe (110), Fe (113), Fe (024), Fe (116) and Fe (112) respectively.^25^ The prominent peaks for GO are located around 13.4, and 23.4 and are assigned to (001) and (002) reflective planes, respectively.^26^ A weak peak of C (001) appears for the case of GO:Fe_2_O_3_ composite with a possible overlap of C (002) and Fe (012) which signifies the formation of the nanocomposite.^27^ The shift in the C (001) peak (13.2° → 13.4°) with the corresponding shift in the C (002) peak positions 23.0° → 24.3° can be attributed to inter-planar spacing originating from the quantity of absorbed water molecules in the carbon matrix.^28^ The shift in peak position is consistent with the shift in the *D*-spacing indicated in Table 1. For the extrapolation of the crystallite size, the full width of the maximum was extrapolated from the Gaussian fit of the consistent 001 and 002 peaks for both GO and GO:Fe_2_O_3_ nanocomposite. The calculated values using the Debye–Scherrer relation^29^ are shown in Table 1. The calculated lattice parameter from the Fe_2_O_3_ dominant phase is *a* = *b* = 5.01 Å and *c* = 13.67 Å, which are comparable with previously reported values.^30^ The absence of lattice parameter for pristine GO is due to the amorphous/least crystalline nature in comparison with Fe_2_O_3_ NP of the C (002) peak.

As indicated in Table 1, there is an increase in the crystallite size of GO when functionalized with Fe_2_O_3_ for both peak positions, suggesting the formation of the composites and the presence of nanosized crystals. A corresponding decrease in the D-spacing is observed for the nanocomposite, which is consistent with broadened FWHM as expected. The XRD spectra are consistent with JCPDS card No. 33-0664.^31^

**Fig. 4:**
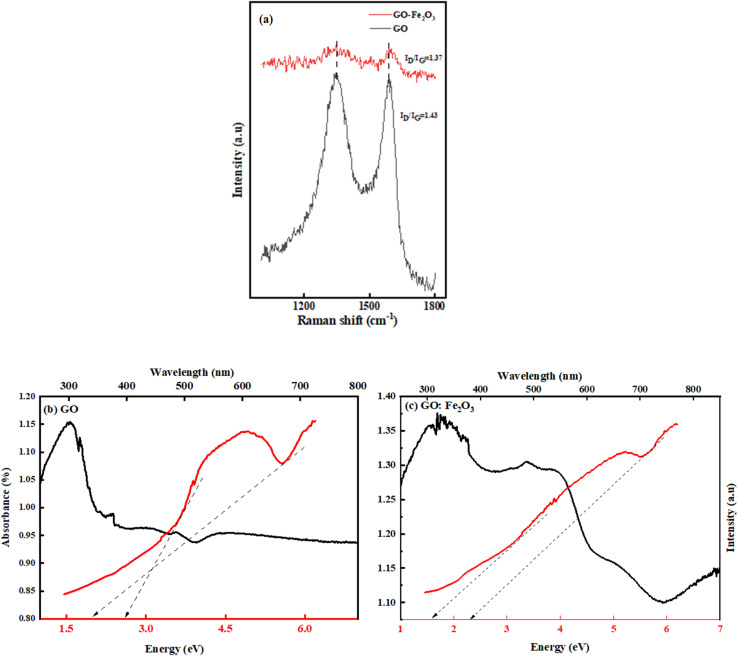
(a) Raman spectroscopy of GO and GO:Fe_2_O_3_ nanocomposite, indicating the *I*_D_/*I*_G_ values. The UV-vis measurement of GO and GO:Fe_2_O_3_ nanocomposite (b) and (c) UV-vis spectra, redline – Tauc plot used for the extrapolation of the energy gap.

Fig. 3(b) shows the FTIR spectra for GO, Fe_2_O_3_ nanoparticles, and GO:Fe_2_O_3_ nanocomposite showing the bonds that are present in the composites. The prominent peaks for GO are located at 1270, 1615, 1732 and 3460 cm^−1^ and are assigned to C–O–C, C=C, C–O and C–OH respectively.^32^ The impact of Fe_2_O_3_ functionalization gives two additional peaks located at 502 and 623 cm^−1^, which are assigned to Fe–O.^33^ The Fe–O peaks are prominent in the Fe_2_O_3_ suggesting Fe-rich nanoparticles with a corresponding decrease for GO:Fe_2_O_3_ nanocomposite. While the appearance of the Fe–O peak signifies the formation of GO:Fe_2_O_3_ nanocomposite, the corresponding decrease in the Fe–O peaks, suggests the formation of C–Fe and Fe–C as established by X-ray photoelectron spectroscopy analysis of our previous report.^34^ The disorder in the defect structure of graphene as indicated by Raman spectroscopy for both samples is shown in Fig. 4(a). The D and G peaks are located at ∼1345 cm^−1^ and 1589 cm^−1^, and show consistency with blueprint features of typical defected graphene.^35^ The G peak is associated with sp^2^ carbon-related materials and is mainly due to C–C bond vibrations. The D band is the defect peak which is due to the formation of vacancies and structural distortions from the graphite exfoliation.^35^ The impact of Fe_2_O_3_ functionalization causes a reduction in the sp^2^ clusters with a broadening of both the D and G peaks. Using the area of both D and G bands, the *I*_D_/*I*_G_ values were calculated. The *I*_D_/*I*_G_ values decrease from 1.43 → 1.37 with a corresponding shift to higher wavenumber (1345 → 1354) and (1589 → 1598), respectively. The increased *I*_D_/*I*_G_ and wavenumber red shift signify a reduction in the oxygen vacancies and Fe atomic attachment on GO.^36^ The broadening of the D and G bands can also be attributed to first-order band broadening which is associated with the graphitic disorder. The band broadening behaviour is consistent with the previous report of Gohel *et al.*^37^

The ultra-violet spectroscopy (UV-vis) which depicts the photo-absorbance properties of GO is shown in Fig. 4(b). The characteristic peak that is associated with GO is located at 207 nm and 302 nm.^38^

The 207 nm peak is attributed to the π → π* C–C aromatic bond transition, whereas the 302 nm peak is mainly due to n → π* C=O bond transitions.^38^ The effect of Fe_2_O_3_ attachment leads to improved absorbance property of GO (0.80 → 1.14) %. The improved absorbance results in the broadening of the n → π* C=O bond transition. The retention of both characteristic peaks signifies the attachment of Fe onto sp^2^ clusters. Based on the Dirac position of pristine graphene, the band gap is expected to be of direct nature, hence the method of Tauc approximation is applicable. Using the Tauc plot analysis, the energy band gap of both GO and GO:Fe_2_O_3_ nanocomposite was approximated. As shown in Fig. 4(b), the Tauc plot indicates two tangential positions resulting in an energy gap (*E*_g_) range of 2.0 → 2.7 eV, which is in proximity with previously reported values.^39^ A reduction in the range of *E*_g_ is observed with Fe_2_O_3_ attachment (1.60 → 2.0 eV) which implies a narrowing of the energy *E*_g_. The value of the obtained *E*_g_ is consistent with the previously reported value.^16^ The report has established the impact of oxidation leads to a variation in the energy gap.^40^ The non-uniform variation in the oxidation content of GO and GO:Fe_2_O_3_ and the double transitions of the π–π and n–π in the absorption account for the variational energy gaps.^41^

**Fig. 5:**
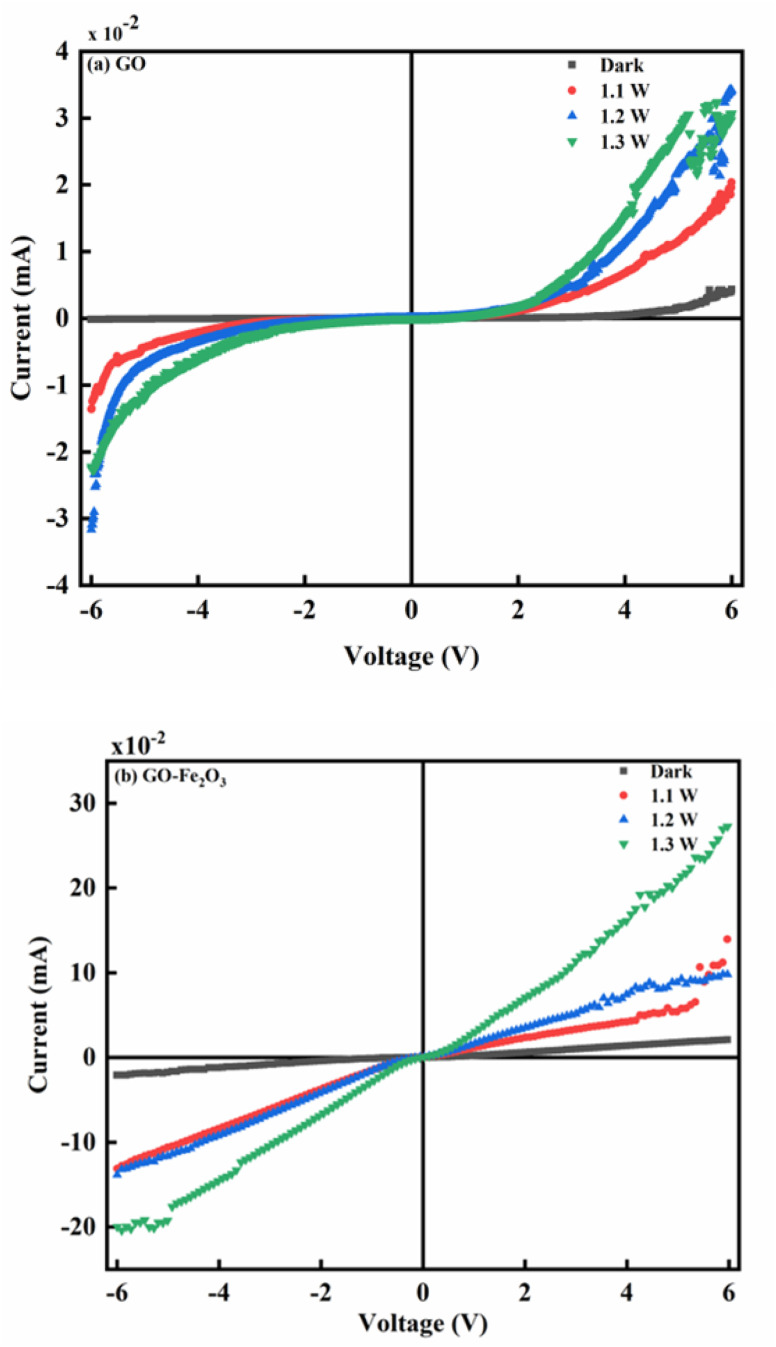
(a): The photo-conductivity measurement of unfunctionalized GO at different illumination intensities. (b): The photo-conductivity measurement of GO:Fe_2_O_3_ nanocomposite at different illumination intensities.

Fig. 5(a) and (b) show the photoconductivity characteristics of GO and GO:Fe_2_O_3_ nanocomposite where the illumination intensity was approximately varied between 1.1 and 1.3 W. Without illumination, the current is independent of reverse voltage and remains independent of forward voltage till ∼4 V when the current starts to increase with voltage, suggesting that GO exhibits a semiconductor behaviour.^18^ An increase in electrical conductivity is observed as the intensity of the light source increases. The increase in the electrical conductivity signifies an increase in the photo-generators, which are responsible for the increased population in the electron–hole (e–h) pair. Reports have indicated the possibility of the e–h generation when the photon energy of the light is greater than the *E*_g_ of the material.^42^ In the current study, the intrinsic plasmon resonance of the graphene matrix coupled with the oxygen functional groups coincides with the selected incident light frequency. The resulting plasmons produce hole e–h pairs through edge scattering with optical phonons.^43^

The impact of Fe_2_O_3_ attachment onto GO results in enhanced photocurrent in the order of ×10 as shown in Fig. 4(b). The attachment of Fe_2_O_3_ causes an increase in the population of e–h pair leading to a more ohmic behaviour. The increase in the e–h pair is mainly due to charge transfer as explained in the work of Shin and Choi.^44^ The attachment of Fe_2_O_3_ onto GO creates a P–N junction characteristic which initiates charge transfer between GO and Fe_2_O_3_. The process continues till there is an overlap between the Fermi level of both GO and Fe. The overlap can be attributed to the mismatch of their work functions.^44^ In addition, there is an accumulation of free charge inherent in the semiconductor which undergoes reduction by the effect of the charge transfer.^45^ The impact of the reduction leads to the generation of the intrinsic electric field. The effect of different intensities of illumination also has an enormous effect on the photocurrent. As can be observed, higher intensity of light gives a corresponding increase in the photocurrent. The enhanced photocurrent can be attributed to the steady generation of photo-generators, which influences the population of electron–hole pairs in the semiconductor. In addition, the intrinsic defects represented by oxygenated groups can be easily excited by the light photons. The generated excitons resulting from the incident light can also contribute to the increased photoconductivity.

The enhanced photocurrent for GO:Fe_2_O_3_ nanocomposite show consistency with the reduced *E*_g_ (1.94 → 1.60) eV that was extrapolated from the UV-vis measurement. A previous report on the photoconductance of bulk^46^ and reduced GO^14,47^ showed enhanced photoconductivity, however, the current study on GO:Fe_2_O_3_ gives improved photoconductivity.

Meanwhile, reports have indicated the distribution of major and minority accounts for the charge storage in electronic devices.^48^ For instance, an n-type semiconductor consists majority of the carrier due to the even distribution of electrons and holes in the respective regions.^49^ Such is the case with our GO sample which exhibits similar behaviour as indicated in Fig. 5(a).

**Fig. 6:**
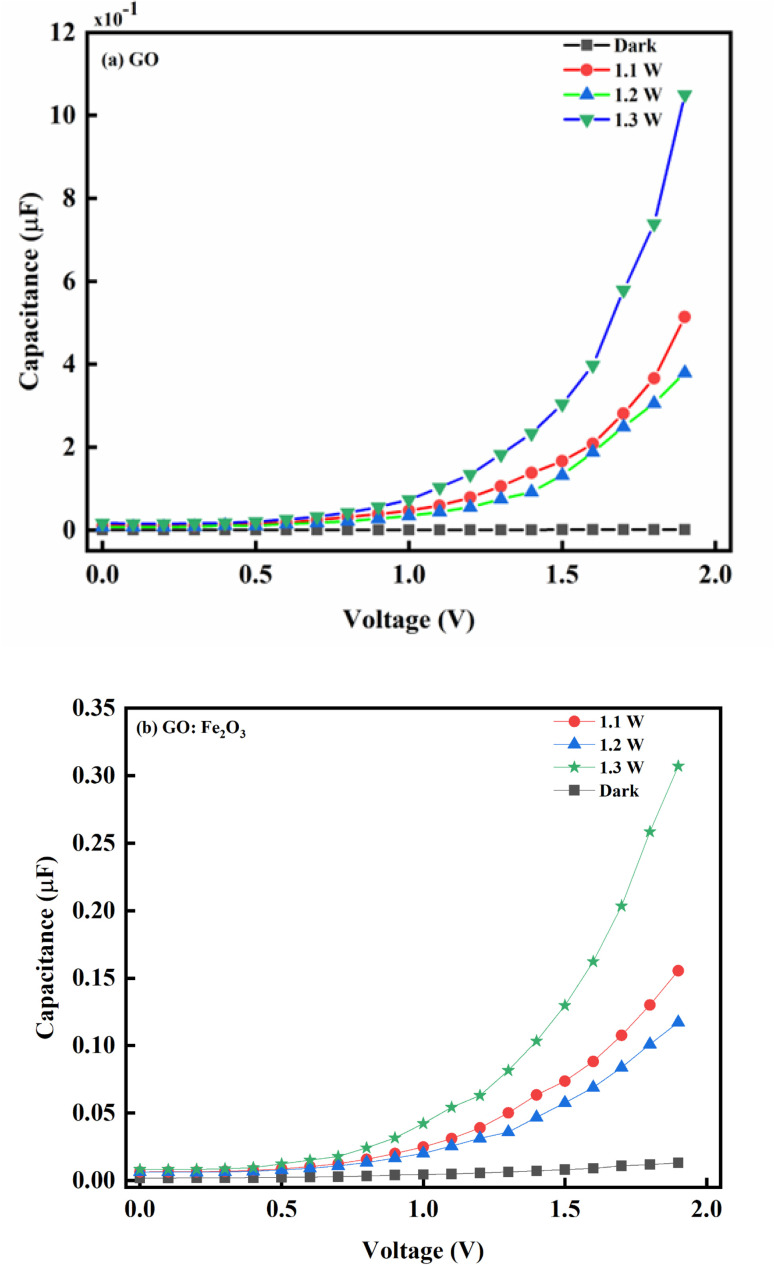
(a): The photo-capacitance measurement of unfunctionalized GO nanocomposite at different illumination intensities. (b): The photo-capacitance measurement of GO:Fe_2_O_3_ nanocomposite at different illumination intensities.

Fig. 6 shows the capacitance–voltage relationship for pristine GO and Fe_2_O_3_ functionalized GO. Consequently, the impact of illumination has a corresponding impact on the behaviour of the charge carrier transport. As indicated in Fig. 6 where there is no illumination, a steady decrease in voltage (2.0 → 0.75 V) with a corresponding decrease in the photo-capacitance and steady saturation till the voltage reaches zero is observed. This charge storage behaviour is consistent with a typical dielectric dipole system where charges are expected to be stored in the charge space layer.^50^ The equality of the reversed and forward voltage bias signifies equal populations of electrons and holes. The impact of light illumination gives a response where the photo-capacitance increases accordingly. The increase in the photo-capacitance with illumination is due to the formation of photo-generators which increases the population of charges being generated. The case of GO:Fe_2_O_3_ composites shows an enhancement in comparison with GO signifying more photosensitivity and charge storage capacity. The charge storage enhancement in GO:Fe_2_O_3_ can be attributed to the formation of charge generation active sites by Fe_2_O_3_ incorporation into the GO matrix.^51^ The corresponding increase in photo-capacitance is also observed when the intensity of the illumination increases.

**Fig. 7:**
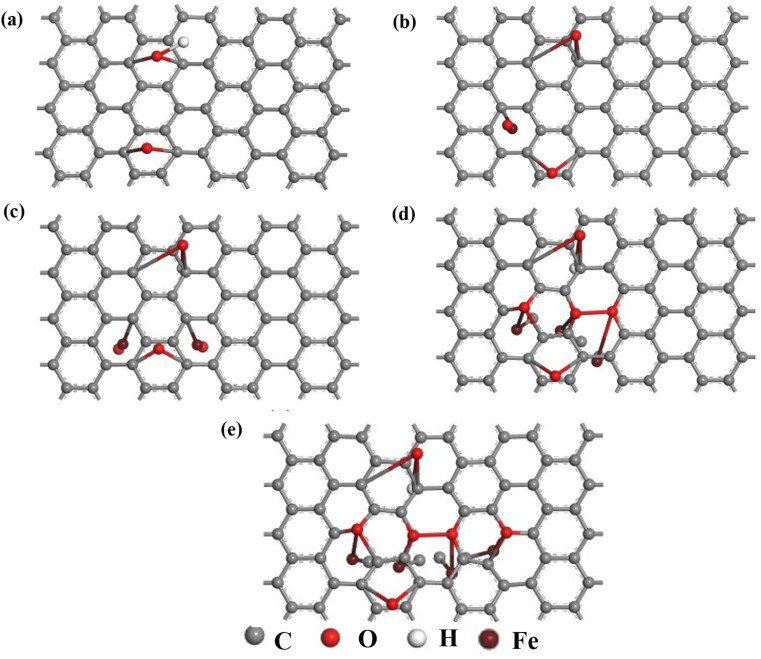
The geometrically optimized structure of undoped and Fe–O doped GO (a) GO (b) GO:Fe–O @ 1 atom (c) GO:Fe–O @ 2 atoms, (d) GO:Fe–O @ 3 atoms and (e) GO:Fe–O @ 4 atoms.

We have further performed DFT calculations to clarify the experimentally observed electronic properties, which account for the photoconductance and photo-capacitance of pristine and Fe_2_O_3_ functionalized GO. The optimized structure for GO and Fe–O doped GO for the four different Fe doping concentrations considered in this study is shown in Fig. 7. After geometry optimization, negligible bond stretching was observed for most of the C–C bonds, signifying easy adsorption and stability of Fe atoms onto the GO matrix.

**Fig. 8:**
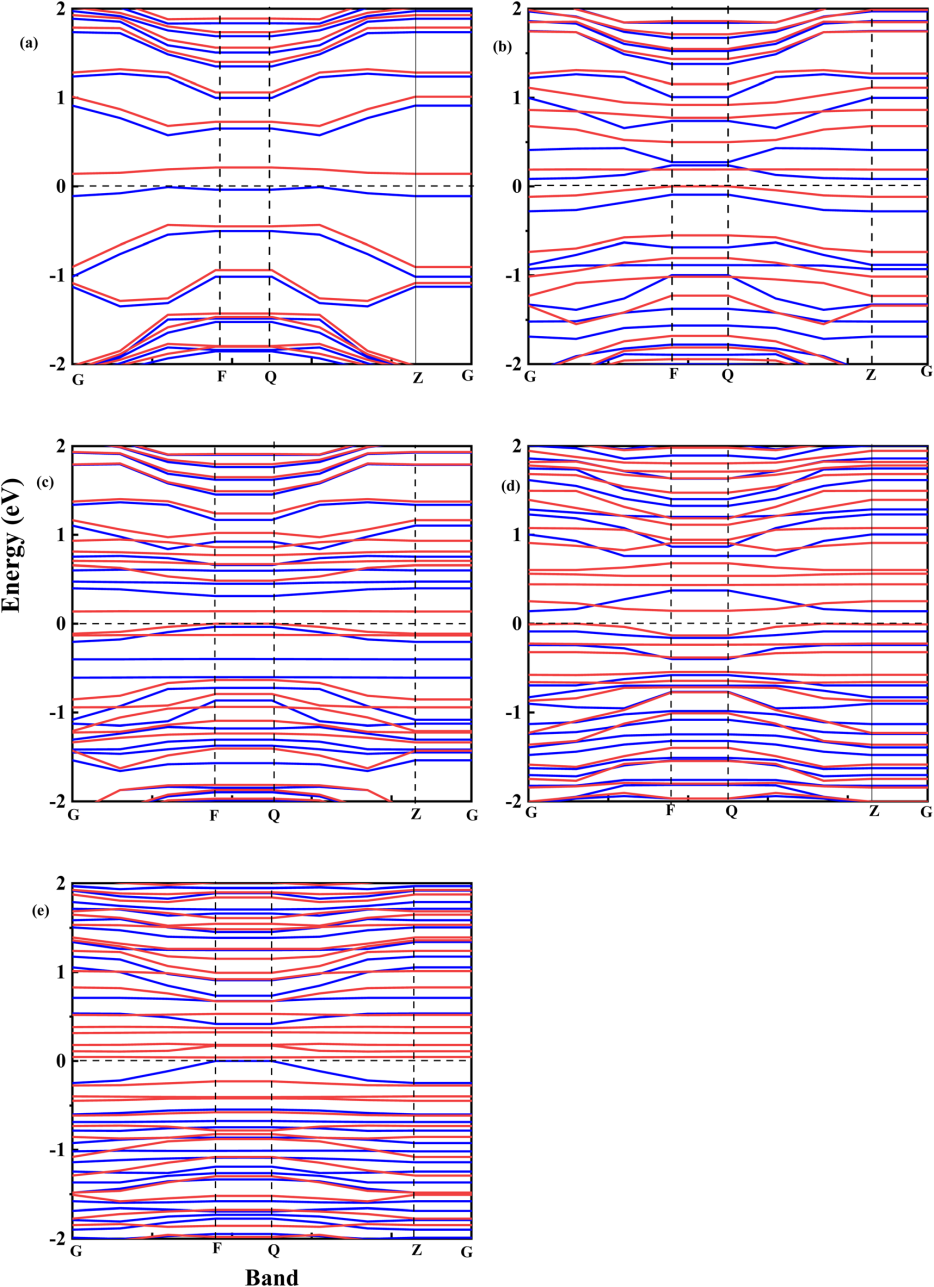
Spin-polarized electronic band structure of (a) undoped GO (b) GO:Fe–O @ 1 atom (c) GO:Fe–O @ 2 atoms, (d) GO:Fe–O @ 3 atoms and (e) GO:Fe–O @ 4 atoms. Red curves represent spin-up band structure while the cyan curves represent spin-down bands, for accurate calculation of the band gap. The horizontal dashed line in each case at 0 eV corresponds to the Fermi level.

Fig. 8 shows the spin-polarized band structure of undoped and Fe–O-doped GO. It is seen that the semiconductor features of GO^52^ are retained after doping with Fe. Based on the calculations of the spin-up (red curve) and spin-down (cyan) curve, the energy gaps were extrapolated.

**Fig. 9:**
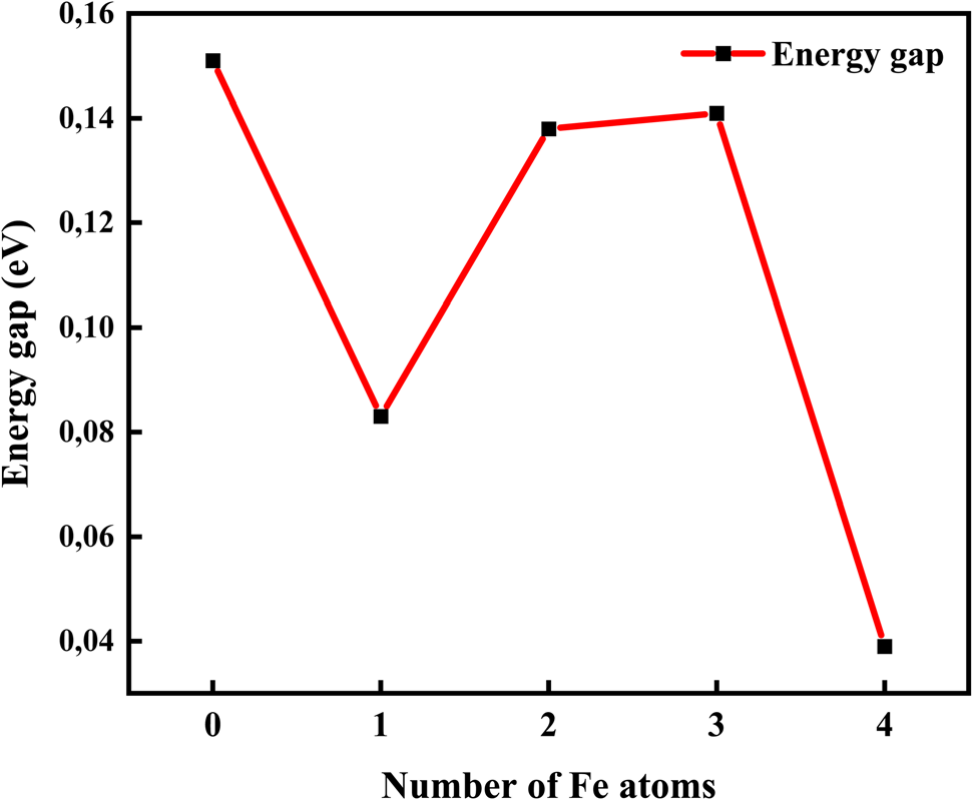
The energy gap opening of GO:Fe–O composite from DFT band structure calculations showing the effect of Fe atom dopant concentration.

Fig. 9 shows the relationship between the energy gap and the number of Fe–O atomic concentrations. The energy gap of undoped GO was calculated to be 0.15 eV, which is in the proximity of previously reported values of single-layer graphene (0.16 eV).^53^ In addition, this energy gap of 0.15 eV of GO is consistent with semi-metal features, which is in alignment with the semiconductor behaviour observed in the experimental data. Doping with one Fe–O atom results in the reduction of the energy gap of GO (0.15 → 0.083 eV), signifying charge redistribution.^54^ Further increase in the number of Fe–O atoms (2 and 3 atoms) leads to the widening of the energy gap (0.083 → 0.138 → 0.141 eV), which can be attributed to charge transfer between C, O and Fe atoms, with Fe–C and C–Fe cluster interaction.^55^ Our earlier report^34^ indicated the formation of Fe–C and C–Fe clusters from X-ray photoelectron spectroscopy analysis in rGO:Fe_2_O_3_ nanocomposite, which is consistent with energy band widening.^56^

Meanwhile, a further increase in the number of Fe–O atoms to 4 results in the narrowing of the energy gap of the GO (0.141 → 0.039 eV), consistent with the experiment data (see Fig. 3(a)). The narrowing of the energy gap leads to the easy transition of electrons around the Dirac points in GO. The narrowing further justifies the increased photoconductance and sensitivity of the photo-capacitance of GO:Fe_2_O_3_ nanocomposite, which was observed in the experimental data.

**Fig. 10:**
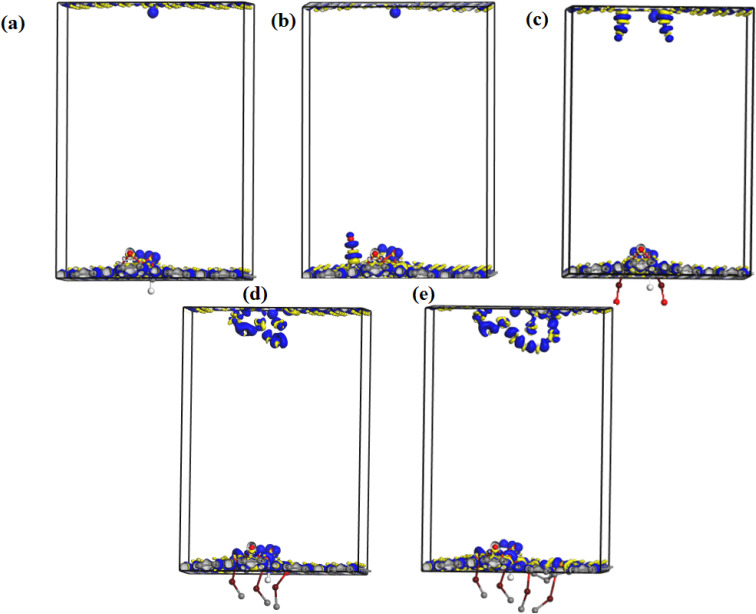
The electron density difference map of undoped and Fe–O doped GO indicating depletion (yellow) and accumulation (blue) regions. (a) Undoped GO (b) GO:Fe–O @ 1 atom (c) GO:Fe–O @ 2 atoms, (d) GO:Fe–O @ 3 atoms and (e) GO:Fe–O @ 4 atoms.

Fig. 10 shows the electron density difference map of undoped and Fe–O doped GO for different Fe–O atomic doping concentrations. The electron density difference depicts the charge transfer/redistribution within the GO matrix. As indicated in the undoped GO configuration, there is an unequal distribution of the charge accumulation and depletion zones. A slight increase in charge accumulation is observed when an atom of Fe–O is introduced in the GO matrix. This increased activity at the accumulation zone can be attributed to charge redistribution thus explaining the reduced energy band gap. The increased Fe–O atomic doping concentration in the GO matrix results in increased activity of the depletion zone with increased accumulation region as expected. The increased accumulation activity can be attributed to the enhanced charge transfer between C, O and Fe with the formation of C–Fe and Fe–O–C clusters.^57^ The increase in the Fe–O atoms (2–3) results in an increased depletion region with insignificant accumulation region activity. The increased accumulation can be attributed to increased charge transfer resulting from strong bonding interactions between C–O–Fe atoms.^58^ The bonding is mostly aided by epoxy/iron oxide interfacial interactions, owing to the strong adsorption through electrostatic interactions.^59^

Further increase in the concentration in Fe–O atoms leads to increased activity in the depletion region. The increased depletion signifies redistribution of the charge within the GO matrix, resulting in increased photoconductivity and sensitivity of the photo-capacitance of GO:Fe_2_O_3_ nanocomposite as depicted in the experimental data.

**Fig. 11:**
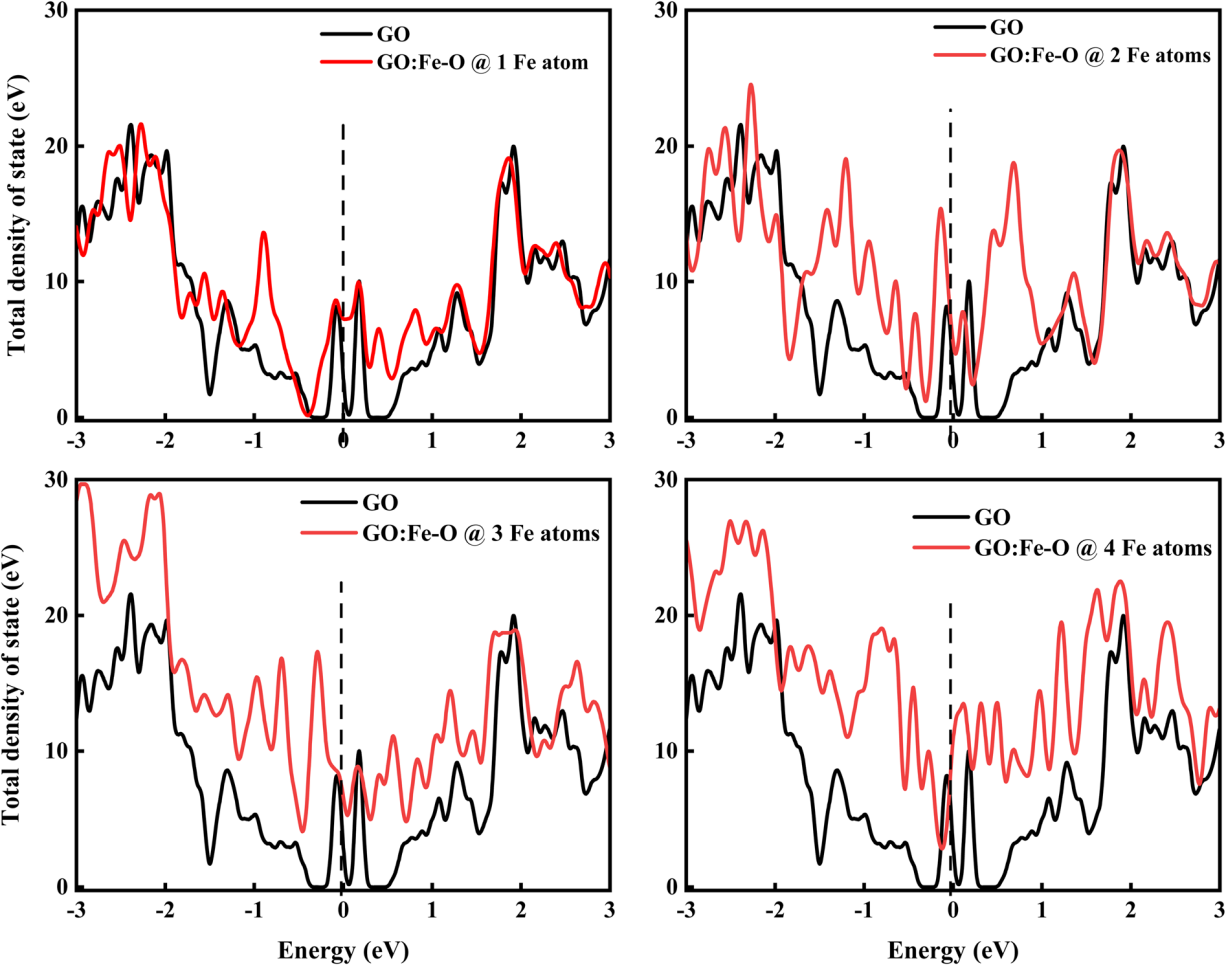
Total density of states of undoped GO and GO doped with 1, 2, 3 and 4 Fe–O atoms. The vertical dashed line at 0 eV in each case represents the Fermi level.

Fig. 11 shows the total density of state of undoped and Fe–O doped GO for different Fe–O atomic configurations. The introduction of 1 Fe–O atom into the GO matrix causes a slight increase in the density of state (DOS) with a slight (0.09 eV) shift below the Fermi level (*E*_f_ = 0) is observed, signifying insignificant formation of states in the GO matrix.^60^ Meanwhile, increased Fe–O (2–3 atoms) leads to the formation of a novel unoccupied state below the Fermi level region, accompanied by a shift below (0.13 and 0.29 eV) the Fermi level and conduction band, respectively, and an increase in the DOS of GO. This result is consistent with strong charge transfer, which is attributed to the increased density of electrostatic potential between graphene and Fe–O interface.^61^

Additionally, the shift of the Fermi level towards the conduction band is consistent with an n-type doped material which signifies an electron-rich with insignificant contributions from electron acceptors.^62^ Further increase of Fe–O doping to 4 atoms lead to an increase in DOS with a shift above (0.1 eV) the Fermi level towards the conduction band. The shift towards the conduction band implies easy attraction of electrons at their defect positions.^63^ The behaviour is consistent with experimental data (see Fig. 4(b)), where the photoconductivity leaned towards a more ohmic behaviour.

**Fig. 12:**
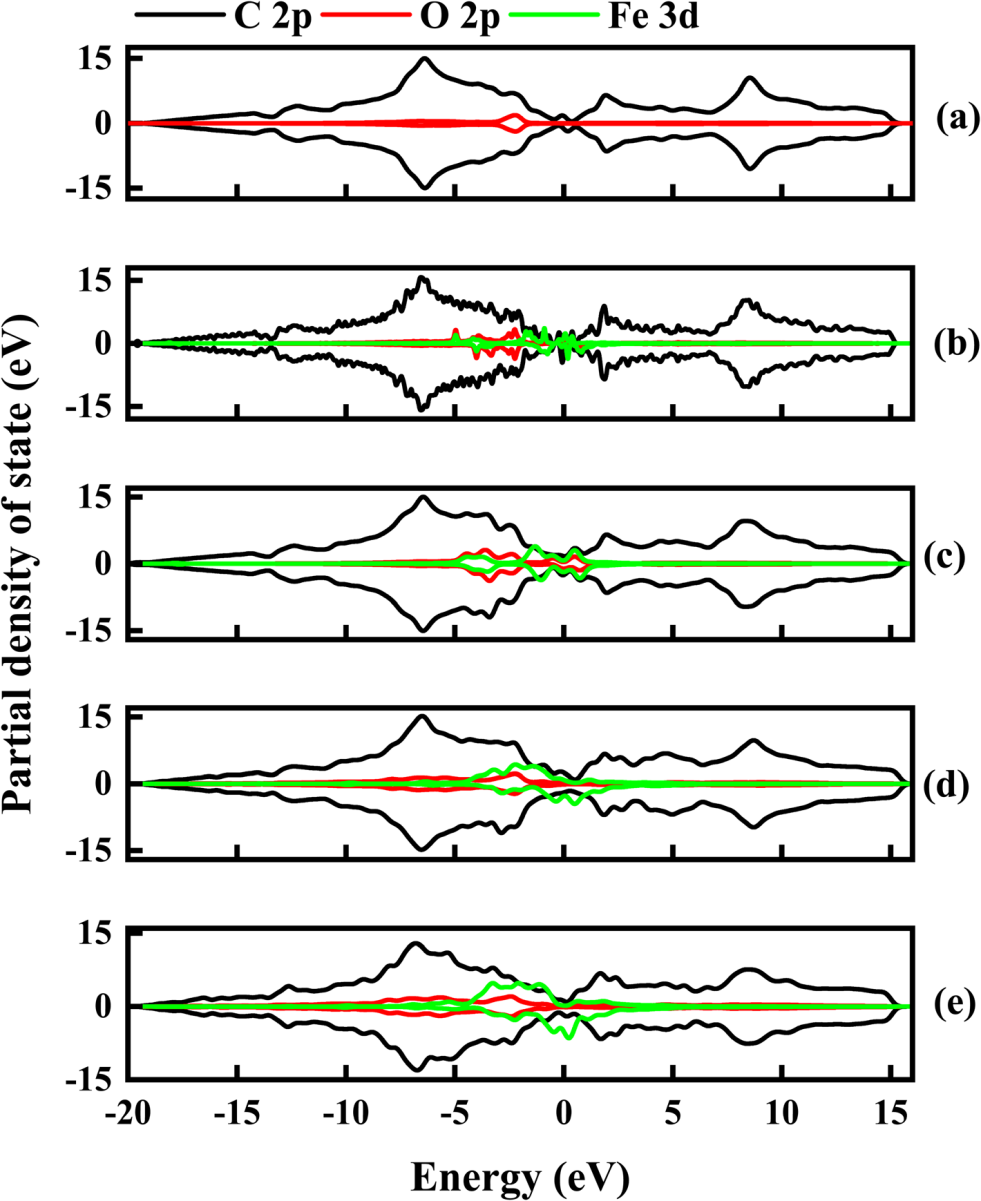
The partial density of state for undoped and Fe–O doped GO. (a) Undoped GO (b) GO:Fe–O @ 1 atom (c) GO:Fe–O @ 2 atoms, (d) GO:Fe–O @ 3 atoms and (e) GO:Fe–O @ 4 atoms.

To further examine the contributions of C, O and Fe in the electronic properties of GO composite, the partial density of state (PDOS) was calculated for all the Fe–O configurations. Fig. 12 shows the spin-up and down PDOS of undoped and Fe–O doped GO. The C 2p orbital states are dominant in all the configurations, as expected, however, a significant contribution is observed for O 2p in the case of GO, which accounts for the widened energy gap of 0.15 eV. The steady increase in Fe 3d is observed with a corresponding decrease in O 2p when Fe is introduced into GO. The increase in the Fe contribution results in increased charge transfer between C and Fe atoms.

**Fig. 13:**
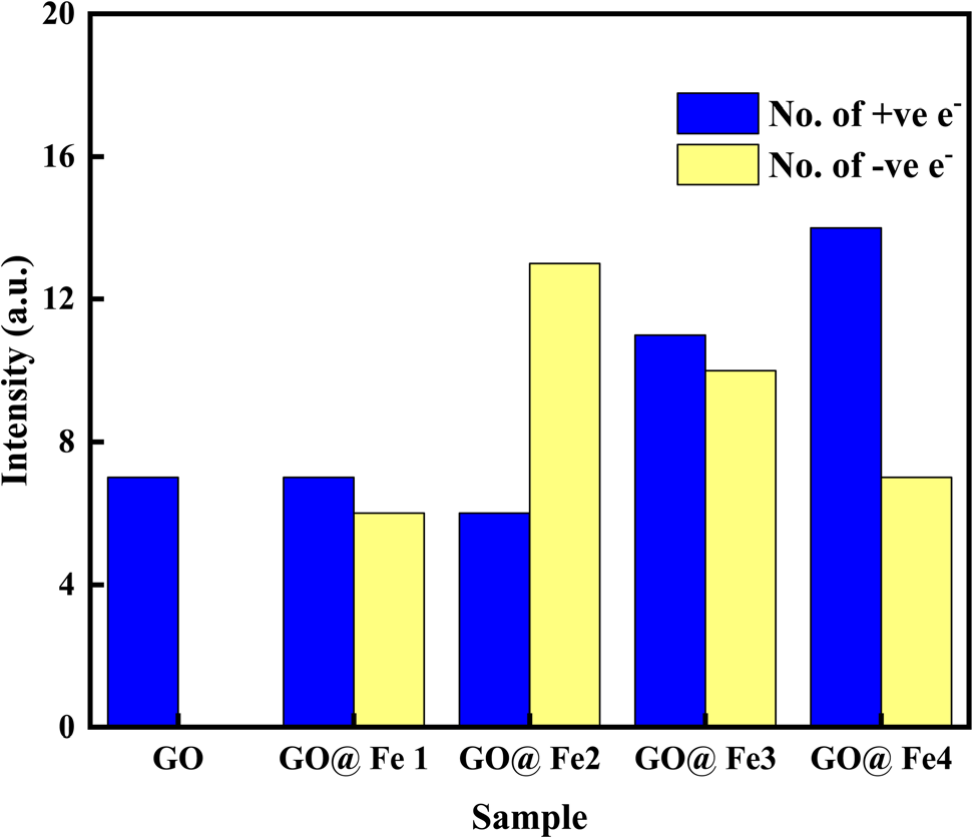
Bar chart showing Mulliken charge analysis of doped and Fe–O doped GO.

Charge transfer is further examined by Mulliken charge population analysis as shown in Fig. 13. Increased electronegativity is observed in low Fe–O doping configuration, signifying the low impact of Fe–O atoms in the GO configuration. However, high doping concentration increases the charge transfer, signifying an increased depletion layer, which is consistent with both the charge density difference image maps and the experimental data.

## 4 Conclusions

In this study, we synthesized GO:Fe_2_O_3_ nanocomposite through a wet co-precipitation technique and studied its photo-conductive and photo-capacitive properties. The results showed an enhanced photoconductivity of the order of ×10 in comparison with pristine GO. An enhanced sensitivity was observed in the case of photo-capacitance. The enhancement is mainly because of charge transfer between Fe_2_O_3_ and GO. The charge transfer leads to the generation of photo-generators, which increases the population of electron–hole pairs in the semiconductor. Density functional theory calculations show the formation of unoccupied states for a high concentration of Fe–O atomic doping. Additionally, increased charge transfer with selective Fe–O doping concentration in GO was observed from electron density difference studies. Hence, it can be postulated that a selective increase in functionalization/doping of GO with Fe nanoparticles can result in charge transfer and overall enhancement in photogeneration. We propose that the GO:Fe_2_O_3_ nanocomposite will find usefulness in the photodetector, photovoltaics, solar radiation detectors and other related optoelectronic device applications.

## Data availability

The data that support the findings of this study are available from the corresponding author upon reasonable request.

## Conflicts of interest

The authors declare no competing financial interests and have no conflict of interest.

## Supplementary Material
